# An efficient green ionic liquid for the corrosion inhibition of reinforcement steel in neutral and alkaline highly saline simulated concrete pore solutions

**DOI:** 10.1038/s41598-020-71222-4

**Published:** 2020-09-03

**Authors:** Mostafa H. Sliem, Ahmed Bahgat Radwan, Farida S. Mohamed, Nasser A. Alnuaimi, Aboubakr M. Abdullah

**Affiliations:** 1 Center for Advanced Materials, Qatar University, 2713 Doha, Qatar; 2 Department of Civil Engineering, College of Engineering, Qatar University, 2713 Doha, Qatar

**Keywords:** Environmental impact, Chemistry, Engineering, Materials science

## Abstract

The effect of the green ionic liquid compound, Quaternium-32 (Q-32), on the corrosion inhibition performance of reinforcement steel, in a simulated concrete pore solution, was investigated at different temperatures and pH values, using electrochemical impedance spectroscopy (EIS). The inhibition efficiency was improved as the concentration of Q-32 and pH values were increased. However, it decreased as the temperature was raised. A Q-32 concentration of 20 µmol L^–1^ exhibited a 94% inhibition efficiency at 20 °C. The adsorption isotherm was evaluated using EIS measurements, and it was found to obey the Langmuir isotherm. The surface topography was examined using an atomic force microscope and scanning electron microscope. The effect of the Q-32 concentration with the highest corrosion efficiency on the mechanical properties of the mortars was also explained by flexure and compression techniques.

## Introduction

Reinforced concrete is utilized in most structures, such as buildings and bridges, because of its excellent service and easy maintenance. Reinforcement steel in concrete structures, especially those exposed to marine and industrial environments, can suffer from corrosion due to many factors, such as pH reduction, carbonation, and chloride attack^1–3^. The chloride content is considered to be one of the main reasons for decreases in the strength of concrete structures. Once the chloride concentration around the steel bar reaches a threshold value, an early structural deterioration takes place^4,5^. The simulated concrete pore solution (SCPS) method is commonly utilized in accelerated tests to investigate the corrosion behavior of the steel in concrete. However, it suffers from some errors due to the heterogeneity in the actual concrete, unlike the simulated one. The common SCPS contains saturated Ca(OH)_2_^6–9^ or cement extract solution^10–13^. It is well known that steel forms a passive layer in SCPS, with a pH ranging from 9.5 to 12. This passive layer becomes unstable and cannot protect it from corrosion when the pH drops below 9.5^14,15^. Several preventive techniques have been proposed and tested to stop or mitigate the corrosion of reinforcement steel. For example, galvanization, epoxy coating, cathodic protection and concrete sealing, in addition to the superplasticizer addition and use of corrosion inhibitors, are among the practices to control the corrosion of reinforcement steel^16–18^. The corrosion inhibitor efficiency in SCPS has been studied by many researchers^19–26^. Most of these studies focus on the adequate dosage of the corrosion inhibitors to provide sufficient inhibition protection for reinforcement steel. However, most of them do not investigate the mechanical properties of the concrete in the presence of the corrosion inhibitor. Corrosion inhibitors in concrete are divided into organic materials, such as alkanolamine and its salts^27,28^, organic acid salt mixtures^29^ and inorganic substances, mainly nitrites. However, nitrites are currently banned in many countries due to their carcinogenicity and biological toxicity^30,31^. Ionic liquids are presented as green chemistry components, which are utilized for clean industrial technology. They are involved in gas capture, fuel cell, chemical synthesis, and catalysts in different reaction process^32–34^. Moreover, ionic liquids with heteroatoms or/and aromatic rings are used as corrosion inhibitors because of their high stability, negligible vapor pressure and eco-friendly nature. Various ionic liquids have been reported to be efficient against corrosion in an acidic medium, but in neutral and slightly alkaline environments, there are insufficient investigations or even essential information to understand the inhibition mechanism of ionic liquid for reinforcement steel. Zhou reported that the corrosion of carbon steel in saline-alkaline solution was reduced by adding 1-butyl-3-methylimidazolium tetrafluoroborate, with an inhibition efficiency greater than 85%^35^. Chong et al. showed that an aprotic imidazolinium cation and 4-hydroxycinnamate anion had a synergistic corrosion inhibition effect on mild steel in neutral, acidic and basic media. The inhibition mechanism is based on the formation of a protective interfacial layer due to the interaction between the steel surface and 4-hydroxycinnamate. Nonetheless, both the components were ineffective on their own, and their combined salt still had an inhibition efficiency of 72%^36^. Besides, sodium aspartate was found to be pH-dependent, with an inhibition efficiency comparable to nitrites. This was attributed to its nature as a diprotic acid. However, sodium lactate showed the lowest ability to inhibit the initiation of pitting corrosion in the presence of chloride alkaline solutions^37^.

This work aims to investigate the effect of isostearyl ethylimidonium ethosulfate [Quaternium-32 (Q-32)] as a green ionic liquid corrosion inhibitor for reinforcement steel in saline SCPS at pH 7, 9 and 12 and temperatures from 20 to 50 °C with the aid of electrochemical impedance spectroscopy (EIS). Changing the pH and temperature and using a saline SCPS is designed to simulate the conditions in which concrete develops over time. Also, the corrosion kinetics and thermodynamic adsorption parameters are measured and/or calculated. Moreover, the effect of adding Q-32, as a component in the cured mortar, on its mechanical properties is reported using the flexural and compression tests.

## Experimental

### Chemicals and materials

**Figure 1:**
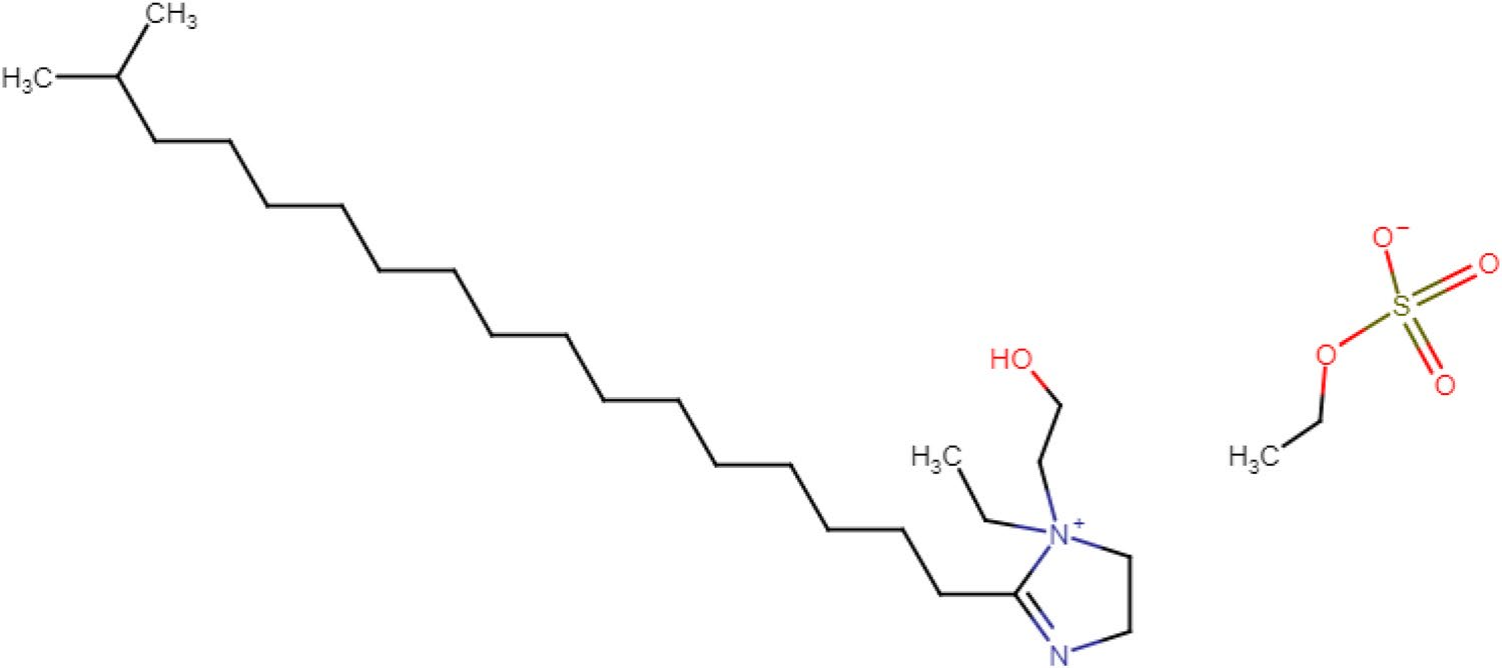
Molecular structure of the Q-32 green ionic liquid corrosion inhibitor.

The samples were cut from reinforcement steel, which was provided by Qatar steel Co., Ltd, Qatar. The steel samples were polished with different grades of silicon carbide form 320-grit to 2000-grit, degreased with acetone, and finally washed with deionized water before being air-dried. The SCPSs were prepared from a saturated calcium hydroxide solution (0.013 M), with 3.5% wt of NaCl adjusted to pH 12^38^. On the other hand, to simulate the carbonation process, NaHCO_3_ powder was added to the solution. Solutions with different pH were used to simulate the cases when CO_2_ reacts with the hydroxides existing in the pore solution to form carbonates and lowers the pH of the pore solution^39,40^. In addition, a standalone 3.5%wt NaCl solution was prepared for corrosion tests at pH 7 to simulate the worst scenario that concrete might face. The pH values of the solutions were verified using a JENWAY pH meter. Ordinary Portland cement obtained from Al Khaleej Cement Company (Doha, Qatar) was used. CEN, EN 196-1 standard sand was purchased from (Société Nouvelle du Littoral). The green ionic liquid inhibitor Q-32 was obtained from Shanghai Dejun Chemical Technology Co., Ltd., Shanghai, China. The chemical structure of the Quenterium-32 ionic liquid is plotted in Fig. 1. Its molecular weight is 520.8144 g, and the molecular formula is C_27_H_53_N_2_O_3_·C_2_H_5_O_4_S (CAS 67633-57-2).

### Electrochemical measurements

Electrochemical measurements were performed in a three-electrode double-jacketed cell. Carbon steel (C-steel) with an exposed area of 0.5 cm^2^ and a graphite rod was used as working and auxiliary electrodes, respectively. An Ag/AgCl electrode was employed as a reference electrode. The reference electrode is coupled with a Luggin capillary to minimize the potential drop between the electrodes. A Julabo F12 thermostat (GmbH, Seelbach, Germany) was utilized to control the temperature of the solutions, as the electrochemical tests were carried out at various temperatures (30, 40, and 50 °C) in the presence and absence of the inhibitor to evaluate the inhibitor efficiency at elevated temperatures. The C-steel was immersed in the test solution for 30 min before each electrochemical test to achieve a steady-state condition. The EIS analyses were performed under an open circuit potential (OCP) condition at a frequency range of 1 × 10^–2^ to 1 × 10^5^ Hz, with an AC amplitude of 10 mV, using a GAMRY 3000 potentiostat (Gamry, Warminster, PA, USA)^41–43^. Echem Analyst Software version 7.8 from Gamry was used to plot and analyze the electrochemical data. For other plotting data, OriginPro 2018 (64-bit) SR1 b9.51.195 was used. Different concentrations of Q-32 (5, 10, 15, and 20) μmol L^−1^ were tested in three different SCPS solutions with different pH values (7, 9, and 12) to investigate its effectiveness in preventing the corrosion of reinforcement steel under different conditions that may develop with time.

### Surface morphological studies

Freshly polished steel coupons were immersed in the different pH SCPS solutions in the presence of 20 μmol L^−1^ of Q-32 for 24 h at a temperature of 20 °C. After that, the coupons were removed, rinsed with deionized water, air dried, then characterized using a high field emission scanning electron microscopy, coupled with an energy dispersive x-ray unit (FEI NOVASEM 450, Hillsboro, OR, USA) in addition to atomic force microscopy (AFM) analysis using an MFP-3D (Asylum Research, Santa Barbara, CA, USA) piece of equipment.

### Mechanical characterization

The effect of using Q-32 on the cured mortar quality was investigated after different exposure times by measuring the changes in compression and flexural strengths, before and after the addition of 20 μmol L^−1^ of the corrosion inhibitor. The compressive experiments were carried out using a 300 KN Tecnotest 3 compression testing machine (Modena, Italy). The flexural strength experiments were utilized by a Lloyd LR 50 K universal testing machine (Ametec Inc. USA). Each experiment was repeated three times, and the results were averaged. The mortar was prepared by mechanical mixing in a stainless steel mixer, with ingredients containing one part mass of cement and one and a half part mass of standard sand, with a water-cement ratio of 0.45^44^. A 20 μmol L^−1^ of the tested corrosion inhibitor was dissolved in the mixing water before applying it into the mortar components. Then, the mold was filled with the mixture under vibration to release air bubbles, and it was stored in a moist atmosphere for 24 h. After that, the demolding of the prepared specimens was conducted, and the specimens were stored under tap water over the test period^45^. The specimens were removed from the water and placed in a drying oven at 60 °C for 24 h before the strength test to avoid the influence of the hydration of the mortar and to increase the strength of the measured specimens^46^.

## Results and discussion

### Electrochemical impedance measurements

**Figure 2:**
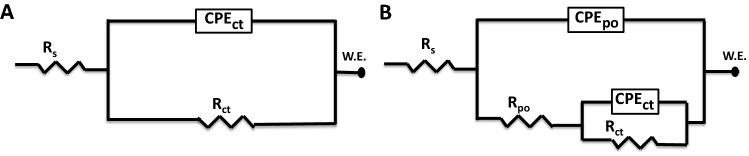
ECs used for EIS analysis.

EIS technique has been employed to describe the electrode/electrolyte interfaces quantitatively. It is considered as a robust method that can explain the corrosion behavior and calculate their rates^47,48^. Figure 2 shows two proposed equivalent circuits (ECs) that are utilized to analyze and fit the collected experimental data. Figure 2A exhibits the one-time constant equivalent circuit, which is commonly used for analyzing electrodes undergoing uniform corrosion. Moreover, the two-time constant equivalent circuit, which is primarily used for electrodes with coatings or adsorbed layers on top^49–51^, is displayed in Fig. 2B. The parameters of the electrochemical reactions occurring at the metal/solution interface are listed in Table1. They are measured and calculated from the EIS Nyquist and Bode plots. From these plots the electrolyte resistance (*R*_s_), pore resistance (*R*_po_), charge transfer resistance (*R*_ct_), constant phase element for the time constant associated with the pore resistance (*CPE*_po_), constant phase elements for the time constant associated with the charge transfer resistance (*CPE*_ct_), and the deviation parameters (*n*_1_ and *n*_2_) from the double-layer capacitance (*C*_dl_) are listed. It is worthy of mentioning that the tests were repeated three times to ensure reproducibility, and the obtained results are the mean value. Additionally, a standard deviation has been tabulated for the crucial parameter such as *R*_ct_ and *C*_dl_.

**Table 1:** EIS parameters for reinforcement steel in SCPS in the presence of various concentrations of the Q-32 corrosion inhibitor with different pH at 20 °C.

C_inh_ µmol L^−1^	R_s_, Ω cm^−2^	R_po_, Ω cm^−2^	*CPE*_po_		R_ct_, Ω cm^−2^	*CPE*_ct_		C_dl_, μF cm^−2^	$\theta$	*IE*, %
			*Y*_po_ × 10^–6^ s^n^ Ω^−1^ cm^−2^	*n*_1_		*Y*_ct_ × 10^–6^ s^n^ Ω^−1^ cm^−2^	*n*_2_			
**pH 7**										
Blank	7.64	–	–	–	290 ± 3.15	830.7	0.593	312.73 ± 2.96	–	–
5	8.56	–	–	–	513 ± 2.39	617	0.576	264.56 ± 2.67	0.434	43.5
10	9.32	–	–	–	712.8 ± 4.87	409	0.677	227.15 ± 4.54	0.593	59.3
15	8.46	–	–	–	1,040 ± 3.92	230.1	0.853	179.84 ± 3.49	0.721	72.1
20	9.29	–	–	–	1,210 ± 2.41	123.7	0.851	88.70 ± 2.72	0.760	76.0
**pH 9**										
Blank	8.26	23.00	822.1	0.816	340 ± 5.13	448.4	0.801	281.03 ± 4.72	–	–
5	7.57	33.71	1,325	0.756	650.5 ± 4.25	359.5	0.764	229.49 ± 4.24	0.477	47.7
10	7.83	37.13	4,143	0.983	898.9 ± 7.84	281.3	0.794	196.90 ± 7.39	0.621	62.2
15	7.95	42.53	4,925	0.530	1,361.8 ± 5.93	148.4	0.876	118.34 ± 5.59	0.750	75.0
20	8.58	51.25	6,036	0.872	2,163.5 ± 6.71	99.5	0.647	43.042 ± 6.33	0.842	84.3
**pH 12**										
Blank	7.897	71.66	989.2	0.924	1,210 ± 4.39	109.1	0.84	74.18 ± 4.14	–-	–-
5	6.380	101.6	719.6	0.599	2,582 ± 8.67	77.19	0.845	57.42 ± 8.13	0.531	53.1
10	7.299	241.2	583.2	0.950	3,990 ± 7.16	47.27	0.799	31.07 ± 6.73	0.696	69.7
15	7.814	481.8	380.5	0.694	7,591 ± 7.42	37.48	0.787	26.67 ± 7.01	0.840	84.1
20	7.938	695.6	119.3	0.841	18,282 ± 6.31	31.15	0.691	24.21 ± 5.95	0.933	93.4

The constant phase element is in place of a pure capacitor as it is composed of the capacitance and deviation parameter to avoid the imperfectness behavior of the ideal double layer, which may occur because of a non-uniform thickness of the corrosion inhibitor layer, non-uniform corrosion reaction on the surface, or non-uniform current distribution and surface roughness^52,53^. The capacitance behavior is mainly attributed to the dielectric nature of the surface film (corrosion product and/or inhibitor film) which affects the corrosion rate of the metal, and it can be expressed by Eq. (1)^54,55^:

1 $$ Z_{CPE} = \left[ { Y_{o}^{ - 1} \left( {j\omega } \right)^{ - n} } \right] $$

where *Z*_CPE_ is the impedance of *CPE* (Ω cm^−2^), *Y*_o_ is a proportional factor in s^n^ Ω^−1^ cm^−2^, *j* = (−1)^1/2^, *ω* is the angular frequency in rad s^−1^, and *n* is the deviation parameter, and its value is between 0 and 1. When n = 1, the *CPE* becomes equivalent to an ideal capacitor, and when *n* = 0, the *CPE* becomes equivalent to a resistor.

**Figure 3:**
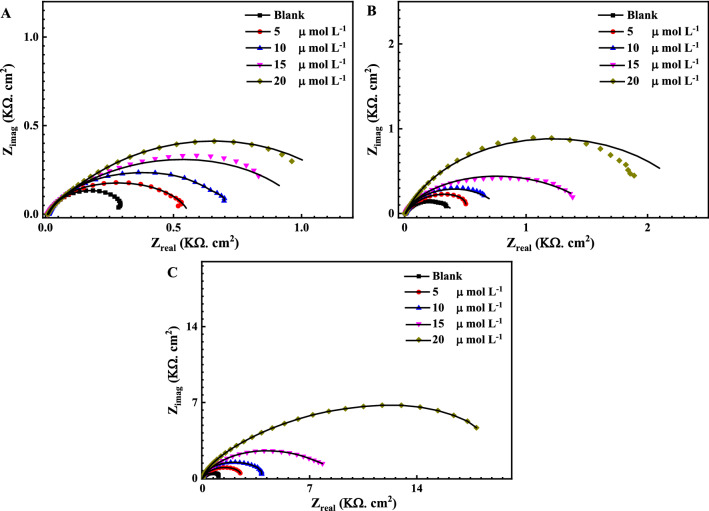
Nyquist plots for the measured EIS data (symbols) and their fittings (solid lines) using the EC shown in Fig. 2 for steel in the presence of different concentrations of Q-32 (5, 10, 15 and 20 µmol L^−1^) and (**A**) 3.5 wt% NaCl solution at pH 7, (**B**) SCPS at pH 9, and (**C**) SCPS at pH 12, within a frequency range of 1 × 10^–2^ to 1 × 10^5^ Hz at OCP.

**Figure 4:**
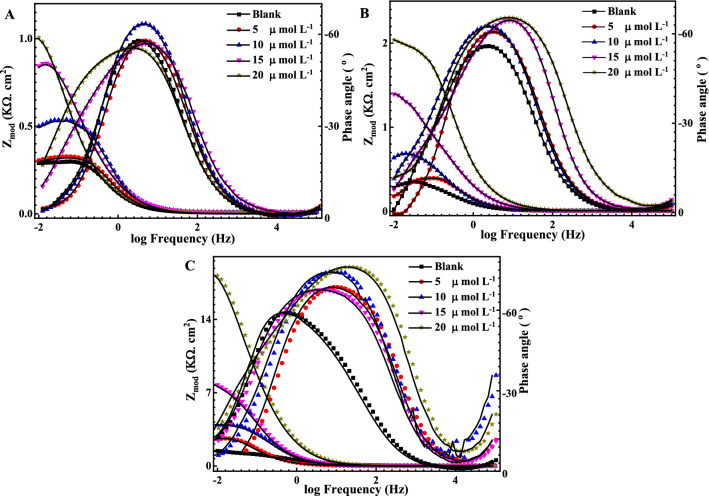
Bode plots for the measured EIS data (symbols) and their fittings (solid lines) using the EC shown in Fig. 2 for steel in the presence of different concentrations of Q-32 (5, 10, 15 and 20 µmol L^−1^) and (**A**) 3.5 wt% NaCl solution at pH 7, (**B**) SCPS at pH 9, and (**C**) SCPS at pH 12, within a frequency range of 1 × 10^–2^ to 1 × 10^5^ Hz at OCP.

Figures 3 and 4 represent the EIS Nyquist and Bode plots , respectively for the reinforcement steel immersed in different pH solutions (7, 9 and 12) at OCP, in the presence of 5, 10, 15 and 20 μmol L^−1^ of the Q-32 corrosion inhibitor at room temperature, within a frequency range of 1 × 10^−2^ to 1 × 10^5^ Hz and at an AC amplitude of 10 mV. The measured data are represented by symbols, while the fitted data, using the equivalent circuits shown in Fig. 2, are represented by the solid lines.

It is evident in Nyquist plots that the higher the pH, the wider the diameter of the semicircle. Besides, a noticeable depression at intermediate frequencies (not perfect semicircles) due to the electrode surface heterogeneity resulting from the roughness of the surface ^56,57^. Furthermore, the width of the phase angle degree curve in the intermediate frequency region of the Bode plot reveals more capacitive response as the capacitive loop diameter increases with the increase in the inhibitor concentration, which means a lower corrosion rate^58–60^. Consequently, the charge transfer resistance (R_ct_) increases proportionally as the corrosion inhibitor concentration increase which is attributed to increasing the surface coverage $\theta$ and the inhibition efficiency $IE_{eis} \%$ of the Q-32 corrosion inhibitor as is introduced in Table 1. The corrosion inhibition is noticeable at the low frequency, and its efficiency increased as the Q-32 inhibitor concentration was increased. This is attributed to the adsorption of more Q-32 molecules at the reinforcement steel surface, which enhances the thickness of the protecting layer on the metal/solution interface^61–63^. It is worth mentioning that the R_ct_ values diminish as the pH value lessens as the R_ct_ values for the reinforcement steel in the presence of 20 mol L^−1^ Q-32 are alleviating from 18.2 kΩ cm^−2^ at pH 12 to 2.1 and 1.2 kΩ cm^−2^ at pH 9 and 7, respectively. The passivity loss of reinforcement steel could be justified for different reasons. It could be attributed to the increase of the Cl^–^ ions adsorption with attenuating the pH, which initiates a passive layer breakdown, thus leading to a localized attack on the metal surface. Additionally, the Cl^–^ ions would penetrate the protective oxide layer leading to form chloride-contaminated oxides. Moreover, the adsorbed Cl^–^ ions would induce the de-passivation potential of the passive film to a value higher than the critical one^64,65^.

The surface coverage $\theta$ and the inhibition efficiency $IE_{eis} \%$ of the Q-32 can be assessed using the *R*_ct_ value from the following relationships^66,67^:

2 $$ \theta = \frac{{R_{ct} - R_{ct}^{o} }}{{R_{ct} }} $$

3 $$ IE_{eis} \% = \theta \times 100 $$

where *R*_ct_ and *R*^o^_ct_ are the charge transfer resistance, with and without the Q-32 corrosion inhibitor, respectively.

It is obvious that the decrease of *C*_dl_ values indicates an increase in the area or the thickness of the electrical double layer. This is attributed to the inhibitor molecule adsorbed on the metal surface, which replaces the adsorbed water molecules. Moreover, the model describes the localized breakdown mechanism of the passive layer, which takes place because of the competitive adsorption of the Cl^–^ and OH^–^ ions, which are inhibited in the presence of Q-32^68,69^. The increase in the charge transfer resistance values may be attributed to either (i) the formed passive film, which is promoted by the presence of the inhibitor molecules that block the active sites on the steel surface, according to Uhlig and Bohni^70,71^, or (ii) the increase in the adsorbed layer thickness/area of the inhibitor, which acts as a physical barrier.

The double layer capacitance (*C*_dl_) is explained according to the Helmholtz model, and can be expressed in Eq. (4)^72,73^:

4 $$ C_{{{\text{dl}}}}  = \frac{{\varepsilon _{0} ~\varepsilon }}{d}{\text{A}} $$

where $\varepsilon_{0}$ is the vacuum permittivity,${ }\varepsilon_{0}$ is the local dielectric constant, *A* is the surface area of the electrode, and *d* is the thickness of the protective layer.

**Figure 5:**
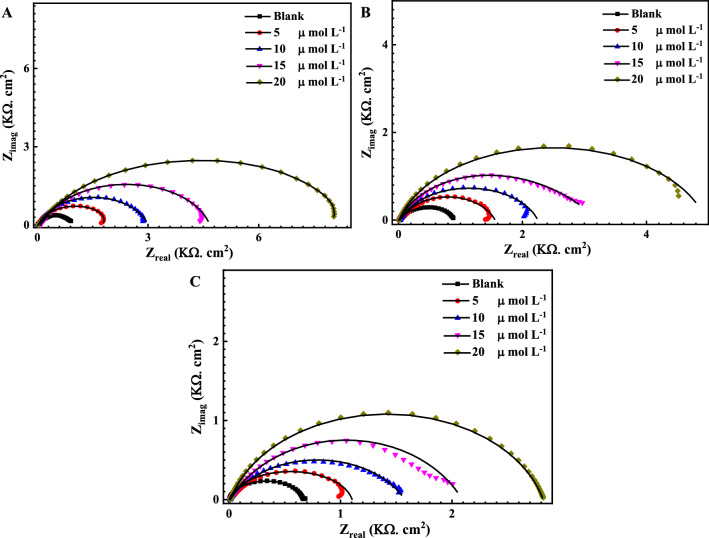
Measured EIS Nyquist plots (dotted) and their fittings (solid lines) for the corrosion of reinforcement steel in SCPS, in the absence and presence of different concentrations of Q-32 and at different temperatures: (**A**) 30, (**B**) 40, and (**C**) 50 °C, within the frequency range of 10 MHz to 100 kHz at OCP.

**Figure 6:**
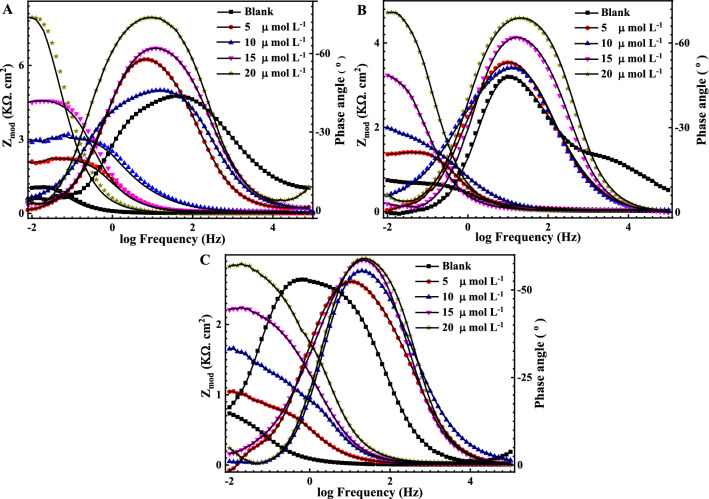
Measured EIS Bode and phase angle graphs (dotted) and their fittings (solid lines) for the corrosion of reinforcement steel in SCPS, in the absence and presence of different concentrations of Q-32 and at different temperatures: (**a**) 30, (**b**) 40 and (**c**) 50 °C, within the frequency range of 10 MHz to 100 kHz at OCP.

Figures 5 and 6 indicate the measured (symbols) and fitted (solid line) of Nyquist and Bode graphs, respectively, for the reinforcement steel after being immersed in saline SCPS, in the presence of 5, 10, 15 and 20 μmol L^−1^ of the Q-32 corrosion inhibitor under elevated temperatures within a frequency range of 1 × 10^–2^ to 1 × 10^5^ Hz at OCP and 10 mV of AC amplitude. Figure 2B demonstrates the two-time constant equivalent circuit, which is deployed for fitting the EIS measurements. It is noticed that for the Nyquist plots, the capacitive loop diameters increase as the inhibitor concentration increases at any temperature. It is also worth mentioning that increasing the temperature reduces the surface coverage as well as the inhibition efficiency. This is attributed to an increase in the desorption rate of the corrosion inhibitor molecules from the reinforcement steel surface, which leads to a surge of the dissolution rate of the electrode surface^19,43^. This indicates that the characteristic features of the EIS measurements do not change as the temperature is altered. A comparison of the *IE*_*eis*_* %* recorded data in Tables 1 and 2 indicates the increasing corrosion inhibition behavior of reinforcement steel with an increasing Q-32 concentration, where the highest inhibition efficiency reached 93.4% at 20 μmol L^−1^ at 20 °C and pH 12. This inhibition efficiency decreases as the temperature increases or the pH and/or Q-32 concentration decrease.

**Table 2:** Electrochemical Impedance Parameters for reinforcement steel in SCPS, in the absence and presence of various concentrations of the Q-32 corrosion inhibitor and under elevated temperature.

T (°C)	C_inh_ µmol L^−1^	Rs, Ω cm^−2^	R_po_, Ω cm^−2^	*CPE*_po_		R_ct_, Ω cm^−2^	*CPE*_ct_		C_dl_, μF cm^−2^	$\theta$	*IE*, %
				Y_po_ × 10^–6^ s^n^ Ω^−1^ cm^−2^	n_1_		Y_ct_ × 10^–6^ s^n^ Ω^−1^ cm^−2^	n_2_			
30	Blank	6.77	52.20	1,570	0.937	974 ± 5.49	183.1	0.802	119.62 ± 5.17	–	–
	5	8.14	85.70	1,190	0.647	1876 ± 6.37	121.6	0.786	81.38 ± 6.01	0.481	48.1
	10	6.48	217.0	813.2	0.877	2,812.8 ± 4.62	77.9	0.859	60.74 ± 4.35	0.653	65.4
	15	4.48	382	567.8	0.589	4,540 ± 7.35	62.9	0.749	41.39 ± 6.92	0.785	78.5
	20	5.97	625	225.8	0.738	8,210 ± 9.02	58.9	0.854	52.01 ± 8.56	0.881	88.1
40	Blank	5.69	62.20	2,989	0.672	850 ± 4.31	218.1	0.801	143.43 ± 4.06	–	–
	5	6.00	77.76	1632.6	0.489	1,490 ± 7.19	154.6	0.848	118.82 ± 6.78	0.429	42.95
	10	4.41	192.3	1,154.4	0.830	2089 ± 5.92	129.3	0.767	86.87 ± 5.51	0.593	59.3
	15	8.49	318.7	839.8	0.465	3,112.2 ± 8.67	98.1	0.748	65.77 ± 8.18	0.726	72.7
	20	6.47	523.4	377.1	0.812	4,845.8 ± 6.79	79.2	0.769	59.45 ± 6.40	0.824	82.5
50	Blank	6.54	72.20	2067.5	0.972	710 ± 4.81	269.7	0.821	188.01 ± 4.53	–	–
	5	4.34	69.34	1724.5	0.679	1,169.3 ± 7.52	239.5	0.723	147.07 ± 7.09	0.392	39.3
	10	5.87	179.8	1,236.0	0.962	1,610.3 ± 8.34	162.6	0.832	124.05 ± 7.86	0.559	55.9
	15	7.18	294.7	859.2	0.557	2069.7 ± 9.13	131.9	0.812	97.66 ± 8.61	0.656	65.7
	20	6.25	493.4	512.6	0.839	2,890.4 ± 5.78	102.3	0.796	74.86 ± 5.45	0.754	75.4

### Inhibitor adsorption and thermodynamic analysis

**Figure 7:**
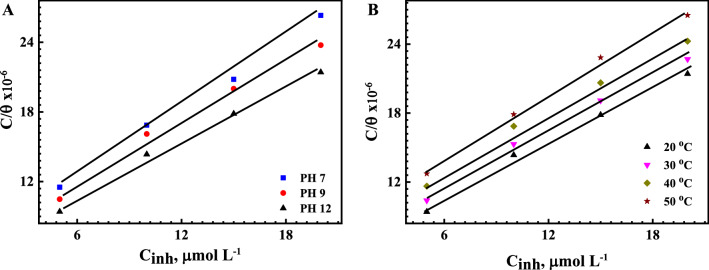
Adsorption isotherm plots for reinforcement steel (**A**) at different pH solutions and (**B**) at saline SCPS, with pH 12 at different temperatures.

The reaction of metal active centers with the corrosion inhibitor molecules occurs via the substitutional replacement process for the electrolyte molecules at the metal/solution interface^74^. The adsorption isotherm models determine the type of reaction, whether it is spontaneously or not, and whether it is physical or chemical interaction from the value of the standard adsorption free energy change (Δ*G*^o^). The relation between $\frac{{C_{{{\text{inh}}}} }}{\theta }$  and *C*_*inh*_ at (A) different pH and B) at different temperatures, which is a straight line, i.e., it follows the Langmuir adsorption isotherm described by Eq. (5), is shown in Fig. 7^75,76^.

5 $$ \frac{{C_{{{\text{inh}}}} }}{\theta } = \frac{1}{{K_{{{\text{ads}}}} }} + C_{{{\text{inh}}}} $$

where *C*_inh_ is the inhibitor concentration, *K*_ads_ is the equilibrium constant of the desorption-adsorption mechanism, and *θ* is the inhibitor surface coverage. A straight-line relationship, with an R^2^ correlation coefficient of 0.98, is shown in Fig. 7. The Langmuir isotherm proposes monolayer adsorption at specific reaction sites on the metal surfaces. Additionally, it is hypothesized that there are no lateral interactions between the adsorbed molecules, and the adsorption is identical and equivalent^75,76^. Meanwhile, the intermolecular interaction between the adsorbed inhibitor molecules, which have donor groups, and the metal surface is not considered in the Langmuir equation, which may cause a small deviation in the calculations^77^. The thermodynamic inhibition mechanism can be utilized to calculate the strength and the type of the adsorption process by calculating the *K*_ads_ values from the intercepts of the plotted straight lines from Fig. 7B. The standard free energy of the adsorption reaction, $\Delta G_{ads}^{o}$, in kJ mol^−1^, can be calculated from with the following equation:

6 $$ \Delta G_{ads}^{o} = - 2.303{ }RT {\log}\left[ {C*K_{{{\text{ads}}}} } \right] $$

where R is the universal gas constant (8.31 J mol^−1^ K^−1^), *T* is the absolute temperature, and *C* is the concentration of water molecules (55.5), expressed in molarity units (M)^78,79^.

The values of *K*_ads_ and *ΔG°*_*ads*_ are given in Table 3. The calculated $\Delta G_{ads}^{o}$ values of the used Q-32 decrease with the increasing pH and temperature. For example, $\Delta G_{ads}^{o}$ at pH 7 is − 38.8 kJ mol^−1^ at 20 °C, and with an elevating temperature at pH 12, the $\Delta G_{ads}^{o}$ values decreased to − 42.2 kJ mol^−1^ at 50 °C. The negative value of $\Delta G_{ads}^{o}$ indicated the spontaneous adsorption of Q-32 molecules on the metal surface. It is reported that, when there are electrostatic interactions between the Q-32 molecules and the charged metal surfaces, the physisorption process is dominant, and the $\Delta G_{ads}^{o}$ values are usually approximately − 20 kJ mol^−1^. Meanwhile, when there is a charge sharing process or electron transfer from the inhibitor molecule to the metal surface to form a coordinated bond, the chemisorption process is expected, and the $\Delta G_{ads}^{o}$ values are near to − 40 kJ mol^−1^. Thus, it can be assumed that both adsorption mechanisms are mainly chemisorbed^80^.

**Table 3:** Thermodynamic parameters derived and calculated based on Langmuir plots.

Temperature, K	*K*_ads_ (L mole^−1^)	$\Delta G_{ads}^{o}$(kJ mol^−1^)	$\Delta H_{ads}^{o}$(kJ mol^−1^)	$\Delta S_{ads}^{o}$(J. mol^−1^ K^−1^)
pH 7	147,000	− 38.8	–	–
pH 9	152,000	− 38.8	–	–
pH 12 (293 K)	170,000	− 39.1	9.81	− 166.98
pH 12 (303 K)	149,000	− 40.1	9.81	− 164.74
pH 12 (313 K)	126,000	− 41.0	9.81	− 162.34
pH 12 (323 K)	119,000	− 42.2	9.81	− 160.90

**Figure 8:**
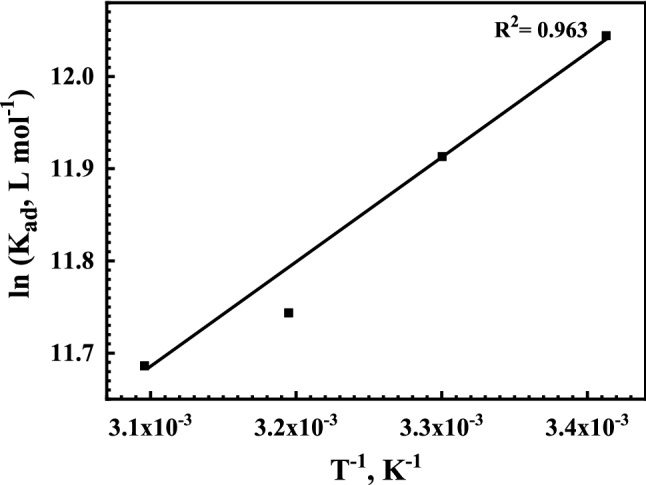
Relation between ln *K*_ads_ and *T*^−1^ of the Q-32 corrosion inhibitor for the reinforcement steel in saline SCPS.

Moreover, the Van’t Hoff equation can be utilized to calculate the standard heat enthalpy change *∆H*^o^_ads_ by plotting a straight-line graph between ln *K*_ads_ versus *T*^−1^, as shown in Fig. 8^59^.

7 $$ {\ln}K_{{{\text{ads}}}} = \frac{{ - \Delta H^{0}_{{{\text{ads}}}} }}{RT} + \frac{{\Delta S^{0}_{{{\text{ads}}}} }}{R} $$

where *∆H*^o^_ads_ and ∆*S*^*o*^_ads_ are the standard enthalpy and entropy changes, respectively.

It is worth mentioning that the standard entropy changes of the adsorption $\Delta S^{0}_{{{\text{ads}}}}$ can be obtained from the intercept of Fig. 8. However, $\Delta H^{0}_{{{\text{ads}}}}$ can also be calculated using Eq. (8), with the help of the calculated standard free energy change of adsorption $\Delta G^{0}_{{{\text{ads}}}}$ and the standard enthalpy changes of adsorption $\Delta S^{0}_{{{\text{ads}}}}$ at different temperatures^81^;

8 $$ \Delta G_{ads}^{o} = \Delta H_{ads}^{o} - T\Delta S_{ads}^{o} $$

### Thermodynamic activation parameters and inhibition mechanism

**Figure 9:**
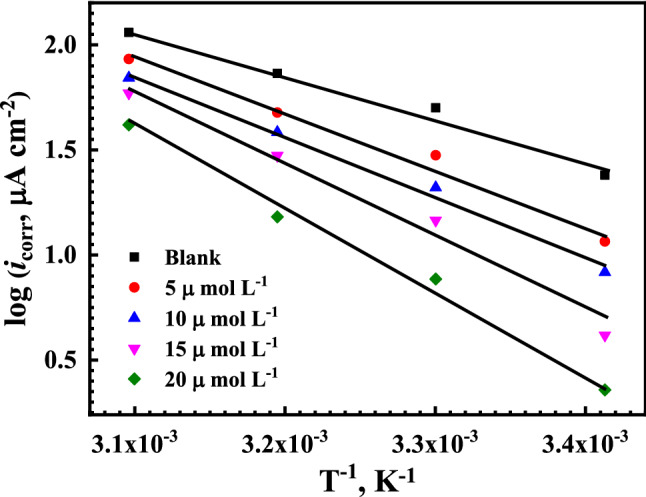
Arrhenius plots for the corrosion current densities (log *i*_corr_) versus 1/*T* for reinforcement steel at different concentrations of the Q-32 corrosion inhibitor in SCPS.

To estimate the activation energy (*E*_a_) for the corrosion of reinforcement steel in saline SCP, without and with the presence of different concentrations of the Q-32 corrosion inhibitor, under an elevated temperature of 20 °C to 50 °C, the relation between log (*i*_corr_) and the reciprocal of the temperature (1/*T*) is plotted to obtain a straight line, as shown in Fig. 9, according to the Arrhenius equation^82,83^:

9 $$ CR = A \cdot exp^{{\left( {\frac{{ - E_{a} }}{RT}} \right)}} $$

where *CR* is the corrosion rate of the reinforcement steel, which is expressed in the (*i*_corr_). *A* is the Arrhenius constant, which varies with the metal type and the electrolyte^84^. The *E*_a_ values are calculated from the slopes of the plotted lines for the relation between log *i*_corr_ and 1/*T*, as shown in Fig. 9, which have a high regression coefficient close to unity, as listed in Table 4. It is shown that the addition of the Q-32 corrosion inhibitor increases the activation energy value, indicating a strong adsorption mechanism on the reinforcement steel surface^85,86^. However, the adsorption of Q-32 molecules on the reinforcement steel surface occurs through both simultaneous chemi/physisorption, as the activation energy parameter refers mainly to the chemical adsorption. This is due to the high competition between the Q-32 molecules and the Cl^–^ aggressive ions and/or the OH^–^ ions for adsorption on the metal surface^87^.

**Table 4:** Activation Energy (*E*a), enthalpy of activation (∆*H*_a_), and entropy of activation (∆*S*_a_) for the reinforcement steel in saline SCPS in the presence of various concentrations of Q-32 corrosion inhibitors.

Conc. of inhibitor µmol L^−1^	*E*a (kJ mol^−1^)	Δ*H*_a_ (kJ Mol^−1^)	Δ*S*_a_ (J mol^−1^ K^−1^)
Blank	40.0	38.19	− 87.23
5	51.0	49.45	− 73.91
10	55.2	53.39	− 63.65
15	68.4	65.84	− 45.17
20	74.0	71.41	− 31.79

**Figure 10:**
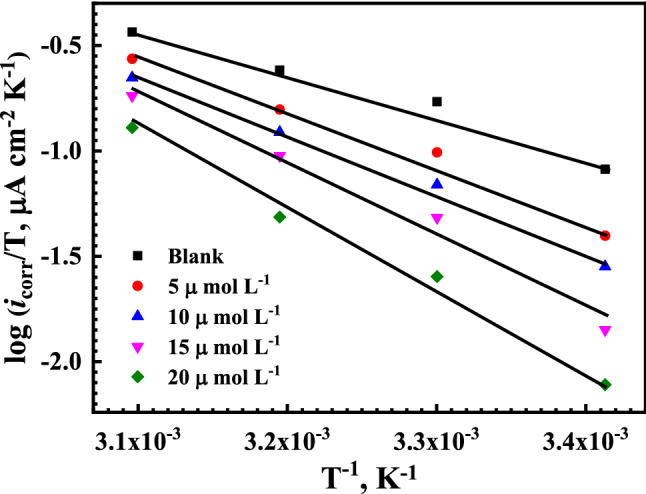
Transition-state plots of log (*i*_corr_/*T*) versus 1/*T* for reinforcement steel in saline SCPS in the absence and presence of different concentrations of the Q-32 corrosion inhibitor.

According to the transition state equation^88^, the values of the apparent enthalpy of activation, Δ*H*_a_, and entropy of activation, Δ*S*_a_, for the reinforcement steel corrosion in SCPS can be calculated from the corrosion rate values (corrosion current density), at different temperatures and in the absence and presence of different concentrations of the Q-32.

10 $$ CR = \left( {\frac{RT}{{N_{A} h}} } \right)\exp^{{\left( {\frac{{\Delta S_{a} }}{R }} \right)}} \exp^{{\left( {\frac{{ - \Delta H_{a} }}{RT}} \right)}} $$

where *h* is the Planck’s constant, *N*_*A*_*,* is the Avogadro's number, and R is the universal gas constant. The plotting of log ($i_{corr} /T$) against $1/T$ gives a straight-line relation, as shown in Fig. 10. The values of Δ*H*_a_ and Δ*S*_a_ are calculated from the slope of the plotted lines and their intercept with the y-axis, respectively, and are tabulated in Table 4. The endothermic nature of the reinforcement steel dissolution reaction is inferred from the positive sign of Δ*H*_*a*_. Increasing the Δ*H*_*a*_ values by adding inhibitors means that the dissolution of reinforcement steel becomes more difficult in the presence of the tested inhibitors^89,90^. The positive trend of Δ*S*_a_ values, referring to the activated complex, is the rate-determining step, which represents an association rather than dissociation, meaning a decrease in the disordering of an activated complex, which is due to reactants^91,92^.

### Scanning electron microscope analysis

**Figure 11:**
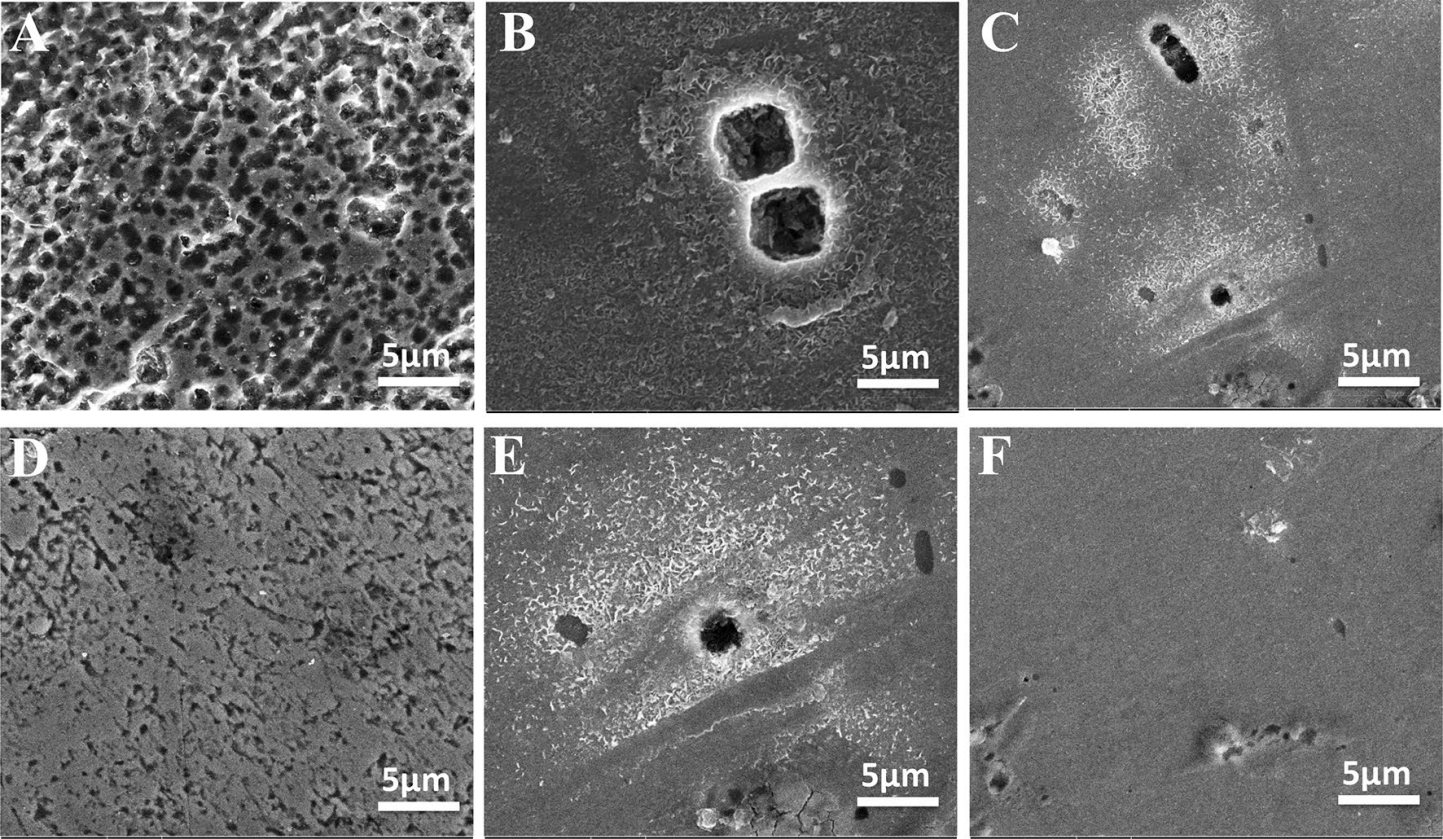
SEM micrographs for the reinforcement steel surface after immersion in saline SCPS for 24 h, (**A**–**C**) in the absence and (**D**–**F**) in the presence of 20 µmol L^−1^ of the Q-32 corrosion inhibitor at pH 7 (**A**–**D**), pH 9 (**B**–**E**), and pH 12 (**C**–**F**) and 20 °C.

The images in Fig. 11 show the surface morphological examination using the SEM micrograph for the reinforcement steel specimens when they are exposed to different pH media in the presence and absence of 20 µmol L^−1^ of the Q-32 corrosion inhibitor, after 24 h of immersion at 20 °C. The steel surface, immersed in 3.5% NaCl, without and with the presence of Q-32, is shown in Fig. 11A,D, respectively. The surface of the specimen is severely damaged and roughened due to highly aggressive media. In the presence of the Q-32 molecules, the corrosion features lessen. The effect of adding 20 µmol L^−1^ of Q-32 to the carbonated SCPS at pH 9 is shown in Fig. 11B,E. The tested sample shows less corroded areas, and a smoother surface can be noticed. Meanwhile, the effect of Q-32 in saline SCPS is shown in Fig. 11C,F, and the protective effect of Q-32 in pH 12, compared to another pH, is notable. The damage of the surface is significantly decreased due to the additional protection obtained by increasing the pH to 12, since the steel surface is passivated at this high pH value.

### AFM analysis

**Figure 12:**
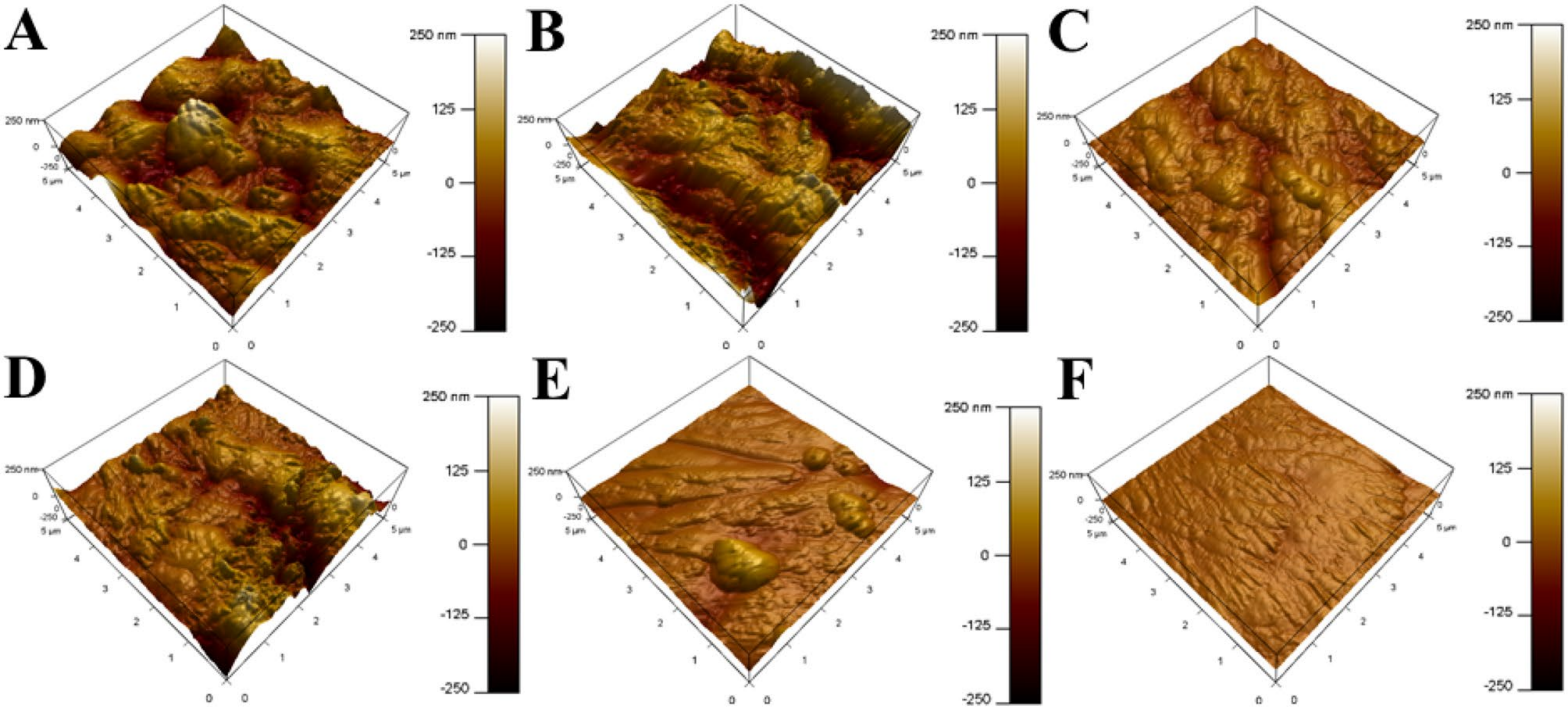
AFM images of the reinforcement steel surface after immersion in saline SCPS for 24 h, (**A**–**C**) in the absence and (**D**–**F**) in the presence of 20 µmol L^−1^ of the Q-32 corrosion inhibitor at pH 7 (**A**–**D**), pH 9 (**B**–**E**), and pH 12 (**C**–**F**) and 20 °C.

AFM is considered a robust technique in the morphological investigation of metal surfaces at the nano-to micro-level and mainly in three-dimensions (3-D). Thus, it can be used to evaluate the activity of the corrosion inhibitor via the surface roughness calculation^79^. The 3-D images of the reinforcement steel surface over an area of 25 μm^2^ are presented in Fig. 12, where the mean roughness factor (*R*_a_) is measured. The surface topography of reinforcement steel, in the absence and the presence of 20 µmol L^−1^ of Q-32 at pH7, is shown in Fig. 12A,D. The *R*_a_ values show that the roughness decreases from 690 to 215 nm in the presence of Q-32 molecules. Meanwhile, the reinforcement steel substrate at pH 9, in the absence and presence of 20 µmol L^−1^ of Q-32, is shown in Fig. 12B,E. The *R*_a_ value at pH 9 is within the range of 430 nm, and with the addition of 20 µmol L^−1^ of Q-32, the *R*_a_ is reduced to 129 nm. Moreover, at pH 12, the *R*_a_ value is 223 nm for the reinforcement steel in the absence of Q-32, which is decreased to 52 nm in the presence of 20 µmol L^−1^ of the Q-32, as shown in Fig. 12C,F.

### Mechanical investigation

The effect of the Q-32 corrosion inhibitor on the cured mortar quality is analyzed using two different mechanical techniques as well as compression and flexural tests. The compression strength was tested on the cubic specimens with dimensions of 50 ×  50 × 50 mm^3^. The flexural strength was tested on rectangular specimens with dimensions of 40  × 40 × 1500 mm^3^. The shown results in Table 5, for the flexural and compressive strength of the cured mortar, after 2, 7 14, 21, and 28 days of exposure in tap water, are obtained according to EN-197-1^93,94^. Cured mortar samples with 20 μmol L^−1^ of Q-32 have a higher flexural and compressive strength, compared to the inhibitor-free ones, for the first 14 days, until it reaches maximum bending stress and breaks at 8.5 MP, after 14 days of the exposure test. Then, the strength remains constant, with no difference due to the presence or the absence of 20 μmol L^−1^ of Q-32.

**Table 5:** Flexural and compressive strength results of prepared cured mortar samples after different exposure times, according to EN-197–1.

Exposure time (days)	Flexural strength Maximum Bending Stress at Break, (MPa)		Compression strength (N mm^−2^)	
	Blank	Q-32	Blank	Q-32
2	5.7	6.4	34.8	41.36
7	5.9	6.7	43.76	44.64
14	6.5	8.3	46.92	46.96
21	6.3	6.5	47.31	47.23
28	6.3	6.3	46.97	47.19

**Figure 13:**
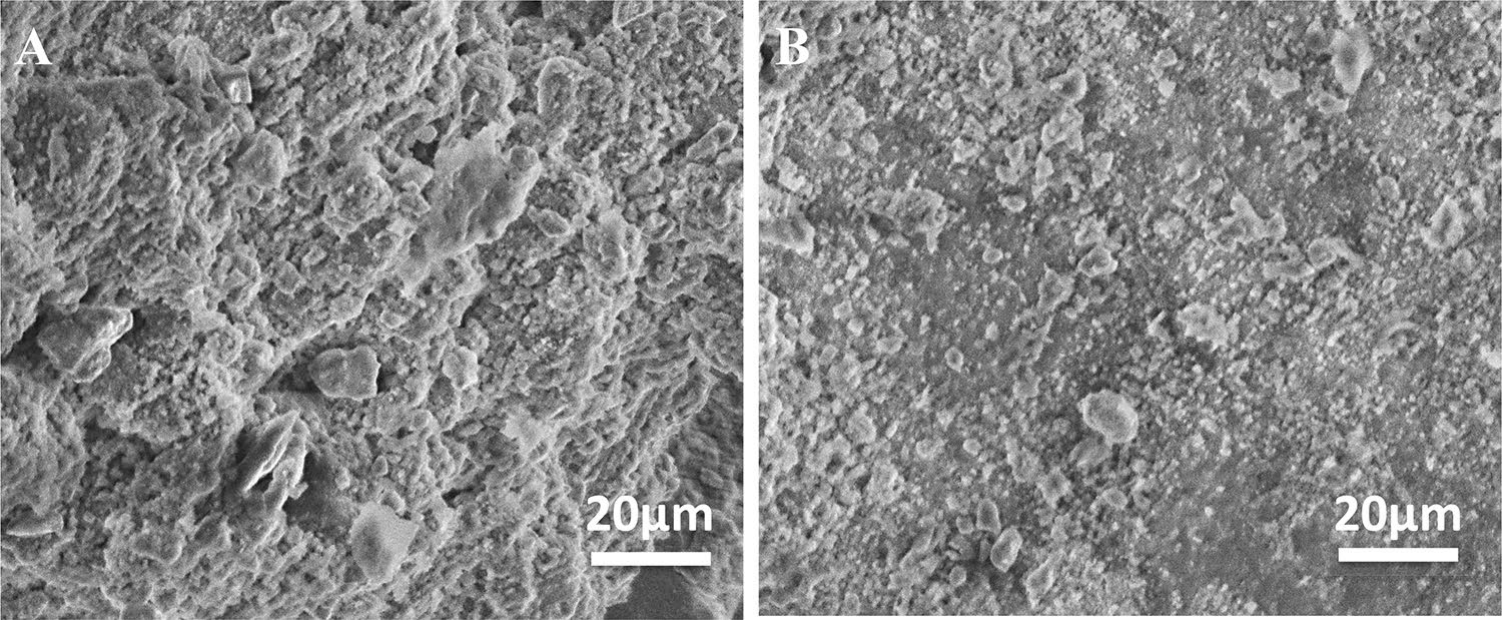
SEM micrograph for the fractured surface of the prepared cured mortar samples (**A**) without and (**B**) with adding 20 µmol L^−1^ after 28 days set.

The SEM images for the fractured cured mortar samples surface in the absence and the presence of 20 µmol L^−1^ after 28 days of age are presented in Fig. 13. The cured mortar surface topography seems to be similar before and after adding the Q-32 corrosion inhibitor to the mixing components, which could be evidence of un reacting the Q-32 corrosion inhibitor with the cement components. It is worthy of mentioning that the cementitious hydration products are represented by the rough surfaces, and the sand particles give a smooth morphology ends^93,94^.

## Conclusions

In this work, the electrochemical results showed that the green ionic liquid, Quaternium-32, is utilized as a corrosion inhibitor for the reinforcement steel in saline simulated concrete pore solution at different pH and temperatures. It is found that the inhibition efficiency *IE*% increases with the increase of the pH and reaches the optimum condition in saline SCPS (pH 12), with 20 μmol L^−1^ of Q-32 at 20 °C. It is confirmed that Q-32 is a chemi/physisorbed process, according to the calculated free energy change values, $\Delta G_{ads}^{o}$. SEM and AFM micrographs for the steel samples immersed in inhibitor-free SCPS depict a high surface roughness, compared to those in the inhibited SCPS. Based on flexural and compressive measurements, Q-32 is suitable for addition to concrete mixtures, with no noticeable effect on the mechanical properties on them after curing.
